# Effect of Freeze Concentration on the Process Parameters, Physicochemical Properties, Total Bioactive Compounds and Antioxidant Capacity of Almond Milk

**DOI:** 10.3390/foods15173021

**Published:** 2026-08-27

**Authors:** Guillermo Petzold, Indira Pérez-Bermúdez, Max Vásquez-Senador, Patricio Orellana-Palma

**Affiliations:** 1 Grupo de Crioconcentración de Alimentos y Procesos Relacionados, Departamento de Ingeniería en Alimentos, Facultad de Ciencias de la Salud y de los Alimentos, Universidad del Bío-Bío, Av. Andrés Bello 720, Chillán 3780000, Chile; max.vasquez2501@alumnos.ubiobio.cl; 2 Centro de Estudios en Alimentos Procesados (CEAP), Avenida Lircay s/n, Talca 3460000, Chile; 3 Escuela de Nutrición y Dietética, Centro Integrativo de Biología y Química Aplicada (CIBQA), Facultad de Ciencias de la Salud, Universidad Bernardo O’Higgins, Calle General Gana 1702, Santiago 8370854, Chile

**Keywords:** plant-based milk, non-thermal technology, solutes, color, total bioactive compounds, antioxidant assays, separation efficiency, solute yield

## Abstract

The objective of this study was to evaluate the application of centrifugal block freeze concentration (CBFC) in fresh almond milk to obtain a concentrated liquid sample with enhanced chemical composition, total bioactive compounds (TBC), antioxidant activity, and process parameters, preserving its physicochemical characteristics. As the cycles progressed, the chemical composition (protein and lipid content), TBC (total polyphenol, flavonoid and protein (albumin) content), and antioxidant activity (2,2-diphenyl-1-picrylhydrazyl (DPPH), ferric reducing antioxidant power (FRAP), and oxygen radical absorbance capacity (ORAC)) significantly increased, reaching 5.2% and 3.3%, 3.16 mg gallic acid equivalents/100 mL, 0.2 mg catechin equivalents/100 mL, 33.5 mg albumin/100 mL, and 55.9, 71.4, and 65.1 µM Trolox equivalents/100 mL, respectively. The separation efficiency and solute yield reached values close to 87% and 72%, respectively. In solute terms, a significant threefold was noted, maintaining an attractive color very similar to that of the fresh sample. Thus, CBFC is an efficient low-temperature concentration method for liquid foods, with lower degradation of TBC than evaporation technology. Hence, the concentrated sample has potential for applications as a functional food and nutraceutical ingredient due to its increased TBC and antioxidant capacity, while its preserved physicochemical stability and chemical composition also suggest feasibility for cosmetic formulations.

## 1. Introduction

Nowadays, the current consumer has updated knowledge about the health benefits of foods, leading to growing interest in varied and healthy diets, favoring the consumption of fruits and fruit by-products, since these foods contain high levels of biocomponents (functional substances, molecules, or materials present in a food matrix that determine its nutritional, functional and physicochemical characteristics, these being crucial for sustainable practices, resource recovery, and health applications, and in turn, they represent a key part of bio-based products in the food and biotechnology industries) [1,2] and high antioxidant capacity [3].

Specifically, the consumption of almonds has increased year to year (1.6 million tons in 2024) [4], and this trend can be attributed to the presence of valuable biocomponents such as proteins, minerals, essential vitamins, unsaturated fatty acids and low sugar content [5,6], and in addition, these fruits are a source of bioactive compounds with antioxidant properties [7]. Furthermore, these fruits contribute to health beneficial effects such as anti-obesity [8], antidiabetic [9], anti-inflammatory [10], anti-aging [11] and anti-cardiovascular [12] activities. Furthermore, the almonds have significant applicability in the food industry due to their sensorial appeal, particularly in culinary and gastronomy aspects, with emphasis on the bakeries and confectioneries areas [13]. However, the almonds have limited seasonal availability (typically from mid-August to October, i.e., the peak of Autumn). Hence, the quality of the fruit in terms of nutritional value, bioactive compounds and antioxidant properties can decrease considerably during post-harvest storage [14,15]. For this reason, many industries have expanded the use of almonds for multiple purposes such as colorants for cosmetics, livestock feed (particularly cattle), production of pellets, compost amended soils, energy biomass, and substrates for cultivation (mushrooms and horticulture), among others [16]. Additionally, the food industry has prolonged the use of almonds for year-round consumption, producing many by-products such as dried fruits, liqueur, jams, ice creams, jellies, yogurt, juices, almond milk, and concentrates [17].

Among almond by-products, in the last decade, the almond milk has been recognized as an alternative to cow’s milk, since almond milk is characterized by a lower energy content than cow’s milk (~30–40 versus ~70–150 kcal per 1 cup (250 mL)), by a low amount of protein, fat (mostly unsaturated versus mostly saturated), carbohydrates and calories, leading to fortification of almond milk with different components such as calcium, vitamin D, and vitamin B_12_, while cow’s milk is characterized by a high content of proteins, calcium, and micronutrients, where the composition depends on fat level (skimmed, semi-skimmed, or whole milk) [18,19]. Additionally, almond milk exhibits poor foaming and coagulation properties and is heat-stable, and its flavor and texture vary considerably depending on the processing methodology used to obtain the final product [20]. Furthermore, in terms of environmental impact, almond milk production generates lower greenhouse gas emissions than cow milk production [21].

Moreover, almond milk preference can be explained by the increasing prevalence of milk allergy and lactose intolerance, as well as the growing adoption of ‘vegan’ and low-calorie diets, and the issues (ethical and environmental) and concerns associated with dairy product production [17,22]. However, almond milk is a highly perishable product, resulting in a short shelf-life, provoking inevitable economic losses during transportation and storage period and adding external factors that significantly reduce the shelf-life of the product such as temperature, oxygen and light, among others [23]. Therefore, the application of innovative technologies could improve the stability of perishable foods and transform them into more stable products which can be stored for a longer period of time, allowing a high retention of biocomponents [24].

Thus, freeze concentration (FC), a non-thermal technology, has generated significant interest in food research as an innovative food processing technique, applicable in various liquid foods, since, in comparison to the traditional thermal process (evaporation), FC allows for concentration at low temperatures. FC is based on water freezing, in which, as the temperature of the liquid food decreases (below its freezing point, avoiding the eutectic temperature), there is a phase separation by a counter-diffusion phenomenon. Hence, the ice crystals expel “impurities” (solutes, vitamins and bioactive compounds, among others) towards their walls, and once the freezing stage is completed, a considerable amount of “impurities” have been rejected from the ice crystals and these “impurities” have been accumulated at the solid–liquid interface. Consequently, two fractions are formed, a concentrated fraction (non-frozen liquid fraction, CC_f_) and an ice fraction (porous solid, IC_f_) [25]. Then, the FC process produces liquid foods with high solute concentration and a remarkable preservation and stabilization of valuable biocomponents [26].

Among FC techniques, block FC (BFC) technique has gained considerable interest in numerous studies, since BFC offers an easy separation step, and a simple equipment and operation procedure. Specifically, in the BFC process, a liquid food solution is completely frozen, and then, the entire frozen solution is thawed, separating the CC_f_ from the IC_f_, since the ice block acts as a porous structure; hence, the CC_f_ moves outwards between the ice crystals [27]. Hence, many studies have demonstrated the successful application of the BFC process to extract and to preserve/retain the characteristics of the initial sample, including BFC studies conducted on maqui [28], blueberry [29], Chilean berries [30], orange [31], pomegranate [32], acerola pulp [33], milk [34], and peppermint infusion [35,36]. These findings highlight the capacity of BFC to significantly improve the retention of bioactive compounds in the separation process. Nonetheless, to the best of our knowledge, no previous studies have investigated the effects of BFC on the properties of almond milk. Thereby, it is essential to conduct a research on the characterization of the CC_f_ from almond milk under centrifugal-BFC (CBFC) in terms of chemical composition (moisture, protein and lipid contents), physicochemical properties (solutes, pH, titratable acidity, and color), total bioactive compounds (TBC) (polyphenols, flavonoids, and proteins) and antioxidant capacity, and in turn, in order to understand the possibilities to apply CBFC technique (45 mL under a freezing stage at −20 °C for 12 h, for, later, a phase separation by centrifugal force at 15 °C for 20 min at 4000 rpm) in almond milk. Moreover, the process parameters (concentration index, separation efficiency, and solute yield) will be analyzed to distinguish the feasibility of scaling up the concentration of almond milk.

Therefore, this study aims to test whether the CBFC treatment at three cycles preserves and concentrates more effectively than thermal evaporation treatment, at the matched total soluble solids content (TSSC) in terms of degrees Brix (°Brix) at each cycle. The comparison focuses on the effects in terms of chemical composition, physicochemical properties, TBC content, and antioxidant activity applied to almond milk. Additionally, for CBFC treatment, separation performance was quantitatively evaluated through the concentration index, separation efficiency, and solute yield.

## 2. Materials and Methods

### 2.1. Materials

Raw almond seeds (*Prunus dulcis* (Mill.) D.A. Webb cv. *Nonpareil*) were obtained from a store located in Parral (36°09′00″ S 71°50′00″ W, Región del Maule, Chile), harvested in the central zone of Chile in the summer of 2025 (Prodalmen Chile, 33°49′00″S 70°45′00″W, Provincia de Maipo, Región Metropolitana de Santiago, Chile). The sample is of 100% purity, i.e., without the presence of other almond varieties or foreign materials. The fruits with uniform color and size, and without any visible surface damage, were selected to obtain the almond milk. Thus, the seeds were collected in plastic bags and transported at the Universidad del Bío-Bío (36°36′10″ S 72°04′38″ W, Región del Ñuble, Chile), and later, the samples were stored at 20 °C until further analysis.

Sodium hydroxide (NaOH), Folin–Ciocalteu phenol reagent, sodium carbonate (Na_2_CO_3_), gallic acid (GA), sodium nitrite (NaNO_2_), aluminum chloride (AlCl_3_), sodium hydroxide (NaOH), catechin (C), Coomassie Brilliant Blue G-250, ethanol, phosphoric acid (H_3_PO_4_), albumin (A), 1,1-Diphenyl-2-picryl-hydrazyl (DPPH), methanol, (±)-6-hydroxyl-2,5,7,8-tetramethyl-2-carboxylic acid (Trolox), sodium acetate (CH_3_COONa), 2,4,6-tris(2-pyridyl)-s-triazine (TPTZ), hydrochloric acid (HCl), iron (III) chloride hexahydrate (FeCl_3_⋅6H_2_O), fluorescein solution, 2,2′-azobis (2-methylpropionamidine) dihydrochloride (AAPH), and phosphate buffer were acquired from Sigma-Aldrich (St. Louis, MO, USA). Distilled water was used throughout the experiment.

### 2.2. Preparation of Almond Milk (AM)

The elaboration of AM was carried out according to the methodology described by Dhakal et al. [37], with slight modifications. Firstly, 100 g of raw almond seeds was soaked in 600 mL of distilled water at 4 °C for 24 h. Later, the soaked almond seeds were drained and rinsed with distilled water, and thus, the skins of soaked almond seeds were manually removed. The soaked almond seeds were ground using a plant-based milk processor (EWSM01 Good Milk, EasyWays SpA, Santiago, Chile) for 5 min at low speed (≈15,000 rpm). Then, the resulting AM was passed through a sieve with a mesh size of 80 mm to remove undissolved solid fragments. Hence, the final filtered AM was transferred into plastic bottles and stored at −20 °C until further analysis. Additionally, commercial AM (CAM) was included as a reference sample to provide contextual comparison with the other samples. The CAM sample was purchased from a local supermarket in Chillán (36°37′15″ S 72°07′20″ W, Región del Ñuble, Chile). Thus, the CAM sample was transferred into plastic bottles and stored at −20 °C until further analysis. Chemical composition, physicochemical properties, TBC content, and antioxidant capacity were determined in both AM and CAM samples.

### 2.3. Concentration Processes

#### 2.3.1. CBFC Treatment

**Figure 1:**
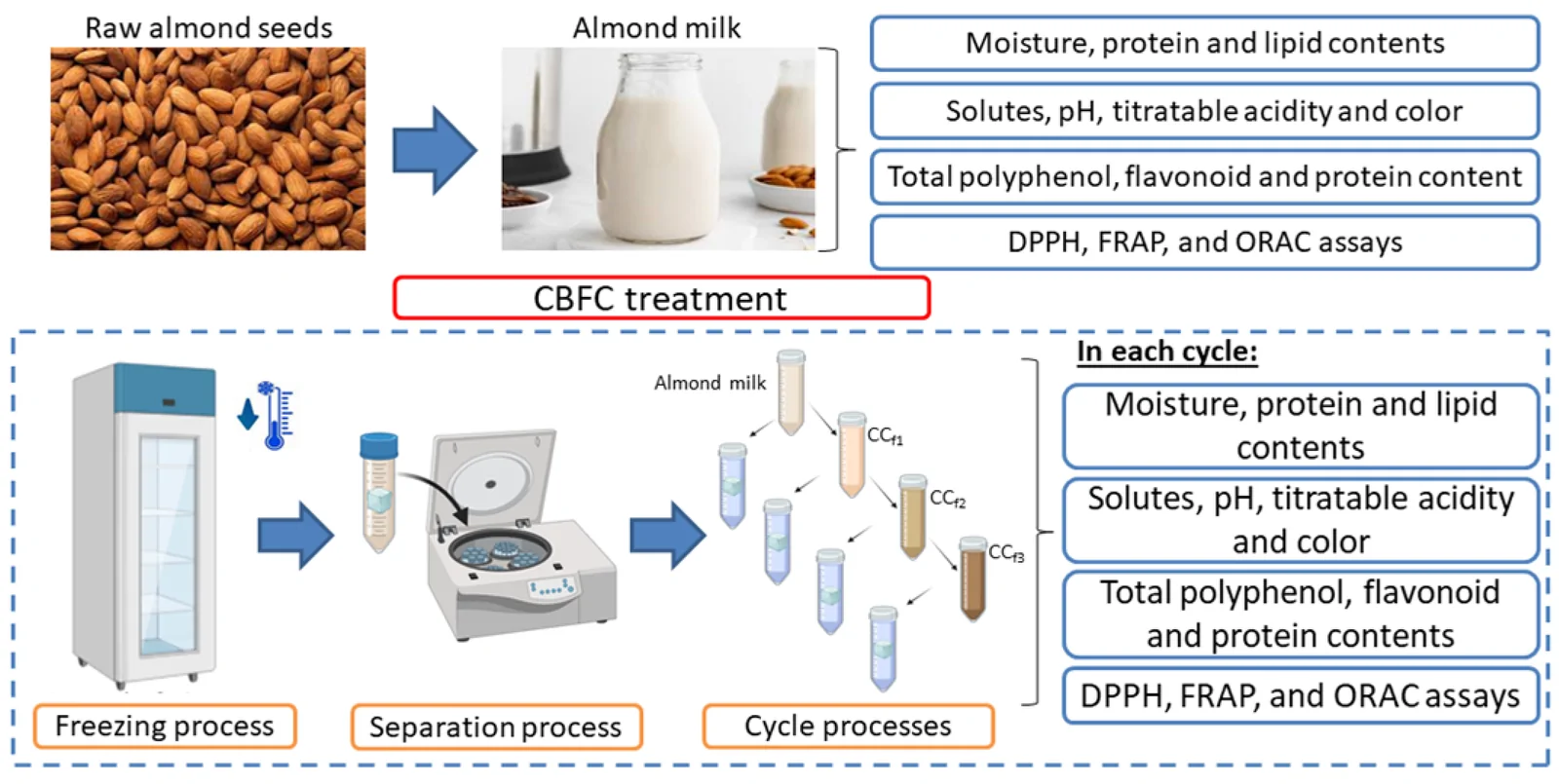
Schematic illustration of the CBFC procedure to obtain concentrates, adding the AM preparation, and each step of the CBFC procedure, i.e., freezing process, separation (centrifugation) step, and each cycle. Additionally, the characterization of AM and concentration can be visualized.

The CBFC procedure was performed by the methodology described by Pérez-Bermúdez et al. [38]. In total, 45 mL of AM was transferred into Falcon tubes, where all the tubes were insulated with thermal polystyrene foam (8 mm thickness, thermal conductivity K = 0.035 W/mK), except in the top and bottom sides, to promote an axial heat transfer during freezing process. Hence, the tubes were frozen in a static freezer (280, M and S Consul, Sao Paulo, Brazil) at −20 °C for 12 h. Later, the tubes were transferred to a centrifuge (Eppendorf 5430R, Eppendorf SE, Hamburg, Germany), inducing the phase separation at 15 °C for 20 min at 4000 rpm. Thus, the CBFC process was performed over three consecutive cycles, where the concentrated fraction (CC_f1_) was extracted from the ice fraction (IC_f1_), and this phase separation corresponds to the first cycle (C_1_). Hence, the 45 mL of CC_f1_ from C_1_ was collected, and it was used as the feed solution in the next (second) cycle (C_2_), where CC_f1_ was frozen (tubes with thermal polystyrene foam) at −20 °C for 12 h, and the phase separation was applied at 15 °C for 20 min at 4000 rpm, and thus, CC_f2_ from C_2_ was again collected for a third cycle (C_3_), using the same operating conditions as the previous cycles (C_1_ and C_2_) in terms of frozen step and phase separation (45 mL under a freezing stage at −20 °C for 12 h, for, later, a phase separation at 15 °C for 20 min at 4000 rpm), obtaining CC_f3_ from the C_3_. The initial volume was 10 L, and after C_1_, the volume in CC_f1_ was 5 L, i.e., approximately 50% of the initial volume in CC_f1_. Meanwhile, the volume recovered in CC_f2_ and CC_f3_ represented approximately 33% and 21% of the CC_f1_ and CC_f2_, respectively. Figure 1 shows the complete CBFC procedure.

The temperature profile in the AM, CC_f1_, CC_f2_, and CC_f3_ was registered at the geometric center of the samples with a thermocouple (type T copper-constantan). The thermocouples were connected to a data acquisition system (CTF84-S8, Ellab A/S, Copenhagen, Denmark) and it registered continuously the temperature in the samples. The freezing rate (μm/s) was calculated as the distance divided by the freezing time (based on the ice propagation rate) [39].

#### 2.3.2. Thermal Treatment (Evaporation)

The thermal treatment was applied to reach a concentrated sample with the same TSSC, in terms of °Brix, as the concentrated solutions at each cycle obtained by CBFC treatment. For evaporation treatment, the concentrated samples were called Ev_1_, Ev_2_, and Ev_3_, having similar concentrations to CC_f1_, CC_f2_, and CC_f3_, respectively. Hence, the AM sample was treated in a rotary evaporator vacuum pump (Rotavapor R-100, Büchi Labortechnik AG, Flawil, Switzerland) at 50 °C for 80 mbar. Thus, chemical composition, physicochemical properties, TBC content and antioxidant capacity were determined in CC_f1_, CC_f2_, CC_f3_, Ev_1_, Ev_2_, and Ev_3_ samples.

### 2.4. Physicochemical Properties

The TSSC was determined using a digital handheld pocket refractometer with an accuracy of ±0.1% Brix (PAL-1, Atago Co., Tokyo, Japan), equilibrating with distilled water at room temperature, and later, 1000 μL of the sample was deposited in the refractometer, and the results were indicated as °Brix. After each measurement, the refractometer was rinsed with distilled water to prevent cross-contamination. The pH was measured using a digital pH meter (HI 2210, Hanna Instruments, Woonsocket, RI, USA), with an accuracy of ±0.01 pH, where before analysis, the pH meter was calibrated with buffer solutions of pH 4.0 and 7.0. The pH of each sample was measured by immersing the probe into the sample and recording the value. After each measurement, the probe was rinsed with distilled water and wiped with a clean tissue. The titratable acidity (TTA) was estimated by the titration method, where NaOH solution (0.1 N) was added to 10 mL of the sample, until persistence (for 30 s) of color similar to a light pink color in the presence of phenolphthalein indicator, and the results were expressed as grams of citric acid (CA) per 100 g of sample (g CA/100 g) [40]. The color was evaluated using a spectrophotometer (Minolta CM-5, Konica Minolta Inc., Osaka, Japan) according to a method previously described by Pérez-Bermúdez et al. [36], with a D_65_ illuminant and a 10° observer angle, where the CIELab parameters (Lightness: L*, from darkness–whiteness axis (from 0 to 100), and two-color coordinates: a*, from greenness–redness axis (from − to +), and b*, from blueness–yellowness axis (from − to +)) were recorded in each sample. Moreover, the degree of color change, also known as the total color difference (ΔE*), between the AM and each concentrated fraction was obtained using Equation (1):

(1) $${∆E}^{*}=\sqrt{{\left(L^{*}-L_{0}^{*}\right)}^{2}+{\left(a^{*}-a_{0}^{*}\right)}^{2}+{\left(b^{*}-b_{0}^{*}\right)}^{2}}$$

where L*, a*, and b* correspond to the CIELab parameters of the CC_f_ in each cycle, while, L*_0_, a*_0_, and b*_0_ correspond to the CIELab parameters of the AM.

### 2.5. Chemical Composition

CAM, AM and concentrated samples were analyzed for moisture, protein, and lipid contents using the methods of Association of Official Analytical Chemists (AOAC) [40]. The moisture content was estimated by placing samples in an oven at 105 °C until a constant weight, and later, the final value was registered. Protein content was determined by the Kjeldahl Method (N × 6.25), where N is the total nitrogen obtained by the method and 6.25 is the conversion factor generally used for almonds and almond by-products used by the Reglamento Sanitario de los Alimentos (RSA) del Ministerio de Salud, Chile. The lipid content was estimated by the acid-hydrolysis method.

### 2.6. TBC Content

#### 2.6.1. Total Phenolic Content (TPC)

TPC was measured by the Folin–Ciocalteu method [41], with slight modifications. In brief, 100 μL of the sample was added to 7900 μL of distilled water, 500 μL of Folin–Ciocalteu reagent (2 M) and 1500 μL of Na_2_CO_3_ (20% *w/v*), mixing the solution for 6 min in an orbital shaker. Then, after 2 h of incubation at room temperature in the dark, the absorbance was measured at a wavelength of 760 nm using a spectrophotometer (T70 UV/vis, Oasis Scientific Inc., Greenville, SC, USA), where the TPC quantification was realized by a GA standard curve, and the results were expressed as milligrams (mg) of GA equivalents (GAE) per 100 milliliters of sample (mL) (mg GAE/100 mL).

#### 2.6.2. Total Flavonoids Content (TFC)

TFC was measured by the aluminum chloride colorimetry method [42], with slight modifications. In brief, 250 μL of the sample was added to 1250 μL of distilled water and 75 μL of NaNO_2_ (5% *w/v*), mixing the solution for 6 min in an orbital shaker. Then, 15,000 μL of AlCl_3_ was added to the solution, mixing the solution for 5 min in an orbital shaker, and later, 500 μL of NaOH (1 M) and 275 μL of distilled water were added to the mixture solution. Hence, the absorbance was measured at a wavelength of 510 nm using a spectrophotometer (T70 UV/vis, Oasis Scientific Inc., USA), where the TFC quantification was realized by a C standard curve, and the results were expressed as milligrams (mg) of C equivalents (CE) per 100 milliliters of sample (mL) (mg CE/100 mL).

#### 2.6.3. Protein Content (PRC)

PRC was measured by the Bradford method [43], with slight modifications. In brief, 100 μL of the sample was mixed with 1000 μL of Bradford reagent (a mixture solution of 5.0 mg of Coomassie Brilliant Blue G-250, 250 mL of ethanol, 5.0 mL of H_3_PO_4_, and 4.75 mL of distilled water). Then, after 30 min of incubation at room temperature in the dark, the absorbance was measured at a wavelength of 595 nm using a spectrophotometer (T70 UV/vis, Oasis Scientific Inc., USA), where the PRC quantification was realized by an A standard curve, and the results were expressed as milligrams (mg) of A per 100 milliliters of sample (mL) (mg A/100 mL).

### 2.7. Antioxidant Activity

#### 2.7.1. 2,2-Diphenyl-1-picrylhydrazyl (DPPH) Method

DPPH method was evaluated according to the methodology used by Brand-Williams et al. [44], with slight modifications. In brief, 150 μL of the sample was added to 2850 μL of DPPH methanolic solution (0.1 mM). Then, after 20 min of incubation at 24 °C in the dark, the absorbance was measured at a wavelength of 515 nm using a spectrophotometer (T70 UV/vis, Oasis Scientific Inc., USA), where the DPPH assay quantification was realized by a Trolox (T) standard curve, and the results were expressed as micromoles (μM) of T equivalents (TE) per 100 milliliters of sample (mL) (μM TE/100 mL).

#### 2.7.2. Ferric Reducing Antioxidant Power (FRAP) Method

FRAP method was evaluated according to the methodology used by Benzie and Strain [45], with slight modifications. In brief, 75 μL of the sample was mixed with 1.5 mL of FRAP reagent (a mixture solution of 25 mL of CH_3_COONa (300 mM, pH = 3.6), 1.3 mL of TPTZ (10 mM in HCl (40 mM)), and 1.3 mL of FeCl_3_⋅6H_2_O (20 mM). Then, after 30 min of incubation at 37 °C in the dark, the absorbance was measured at a wavelength of 593 nm using a spectrophotometer (T70 UV/vis, Oasis Scientific Inc., USA), where the FRAP assay quantification was realized by a Trolox (T) standard curve, and the results were expressed as micromoles (μM) of T equivalents (TE) per 100 milliliters of sample (mL) (μM TE/100 mL).

#### 2.7.3. Oxygen Radical Absorbance Capacity (ORAC) Method

ORAC method was evaluated according to the methodology used by Ou et al. [46], with slight modifications. In brief, 30 μL of the sample was added to 20 μL of fluorescein solution (10 nM), and the solution was deposited into black 96-well microplates and incubated at 37 °C for 30 min. Thus, the solution was added to 50 μL of AAPH (600 mM) and 2900 μL of phosphate buffer (75 mM), and the absorbance was measured at a wavelength of 485 nm and an emission set of 520 nm, where the procedure was made every 1 min for 60 min, using a multimode plate reader (Victor X3, Perkin Elmer, Hamburg, Germany), and hence, the ORAC assay quantification was realized by a Trolox (T) standard curve, and the results were expressed as micromoles (μM) of T equivalents (TE) per 100 milliliters of sample (mL) (μM TE/100 mL).

### 2.8. Sensory Evaluation

Four samples (CAM, AM, and samples concentrated by CBFC and concentrated by evaporation from the third cycle) were reconstituted to 1.1 °Brix (TSSC value of CAM) by adding the appropriate amount of water. The reconstituted samples were evaluated by a trained sensory panel composed of 21 panelists (ten males and eleven females, with an average age of 30 years). Hence, the samples were rated according to a 5-score hedonic scale system, ranging from 1 (I dislike it very much) to 5 (I like it very much). Specifically, odor, aroma, flavor and overall acceptability were evaluated. The samples were served in 25 mL plastic cups, containing 5 mL of sample, at room temperature, where each cup was randomly coded with a three-digit number. Cold water and soda crackers were provided to each panelist for rinsing their palates between each sample. Additionally, before the test, all the panelists were informed about the study, and they signed an informed consent form to undergo the sensory evaluation.

### 2.9. Process Parameters

#### 2.9.1. Concentration Index (CI)

CI corresponds to the increase in solutes of the CC_f_ in each cycle with respect to the initial solutes of the AM. The calculation formula (Equation (2)) is as follows [35]:

(2) $$CI=\frac{{SCC}_{f}}{{SCC}_{0}}$$

where SCC_f_ is the solute in the concentrated fraction (CC_f_) in each cycle and SCC_0_ is the initial solute of the almond milk (AM).

#### 2.9.2. Separation Efficiency (Eff_s_)

Eff_s_ corresponds to the rise in solutes of the CC_f_ in each cycle with respect to the solutes of the IC_f_ in each cycle. The calculation formula (Equation (3)) is as follows [28]:

(3) $${Eff}_{s}=\left(\frac{{SCC}_{f}-{SIC}_{f}}{S{CC}_{f}}\right)\times 100\%$$

where SCC_f_ is the solute in the concentrated fraction (CC_f_) in each cycle and SIC_f_ is the solute in the ice fraction (IC_f_) in each cycle.

#### 2.9.3. Solute Yield (S_y_)

S_y_ corresponds to the ratio between the mass of solutes of the CC_f_ in each cycle with respect to the initial mass of solutes of the AM. The calculation formula (Equation (4)) is as follows [39]:

(4) $$S_{y}=\left(\frac{{CC}_{f}\cdot m_{f}}{C_{0}\cdot m_{0}}\right)\times 100\%$$

where m_f_ is the mass of solutes of the concentrated fraction (CC_f_) in each cycle and m_0_ is the initial mass of solutes of the almond milk (AM).

### 2.10. Statistical Analysis

The data were obtained through three independent experiments, where all analyses were performed in triplicate. The results were shown as mean ± standard deviation. The data were submitted to a one-way analysis of variance (ANOVA) to identify differences between samples, and a least significant difference (LSD) test was conducted for the comparison between pairs of means (*p* ≤ 0.05). Statgraphics Centurion XVI Software (v. 16.2.04, Statpoint Technologies Inc., Warrenton, VG, USA) was employed for statistical analysis.

## 3. Results and Discussion

### 3.1. Freezing Curve

**Figure 2:**
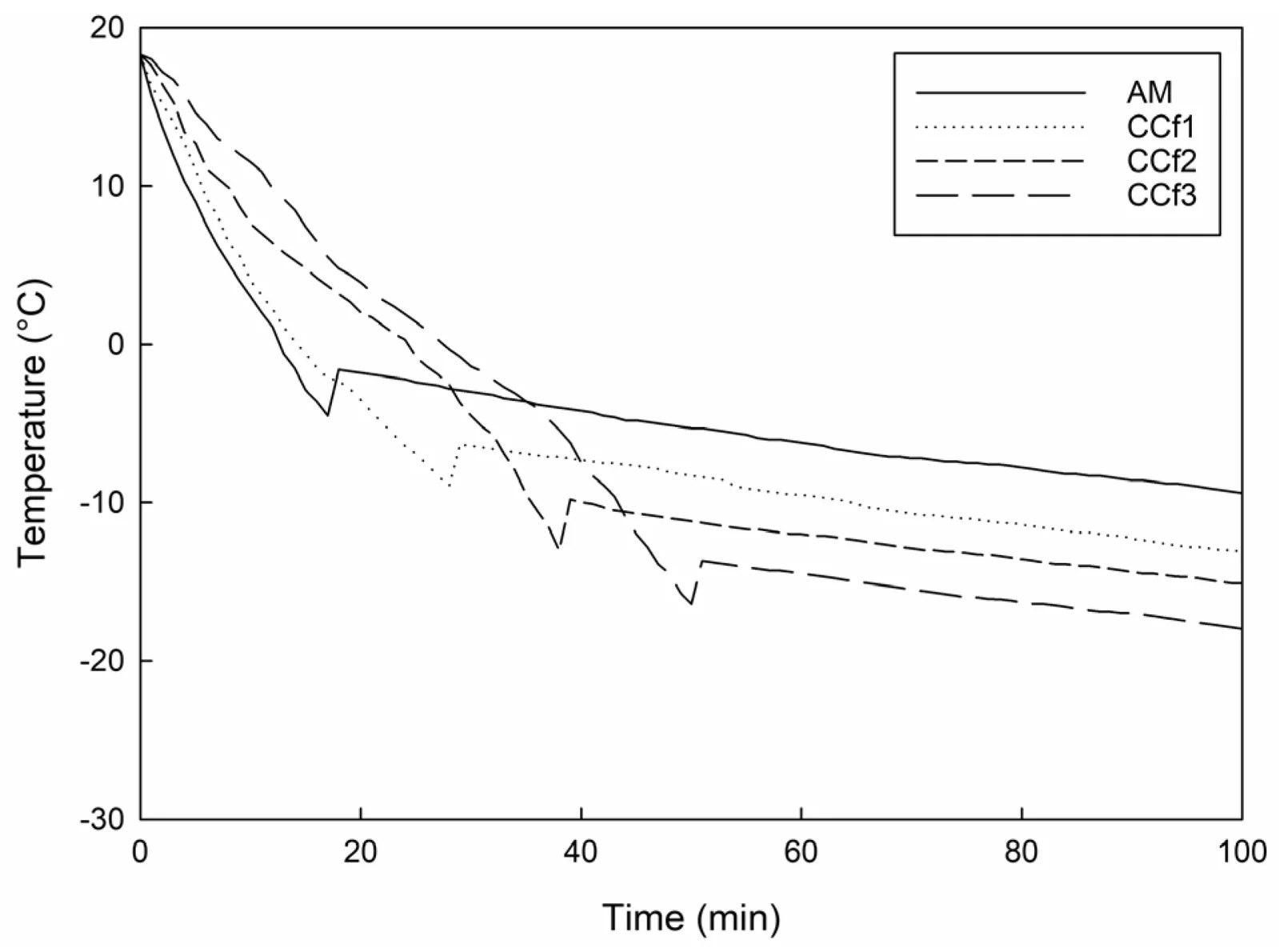
Freezing curve of the samples.

Figure 2 shows the freezing curve of the samples (AM, CC_f1_, CC_f2_, and CC_f3_).

Firstly, it is possible to denote clear differences in freezing point depression among all the samples, particularly in the freezing plateau observed between AM and each concentrated fraction. The initial sample (AM) showed a freezing point depression value close to −2.0 °C, and its value was higher than that reported for commonly consumed food products such as blueberry juice, with a freezing point depression value close to −3 °C [47], since blueberry juice contains a higher concentration of sugars and organic acids than almond milk [48]. In the same way, wine (an alcoholic beverage) shows a freezing point depression value of approximately −5 °C [49], which is associated with the presence of ethanol, a compound with a much lower freezing point than water [50,51]. Additionally, CC_f1_, CC_f2_, and CC_f3_ presented freezing point depression values close to −6.0, −10.0, and −14.0 °C, respectively. This effect can be explained by the water removal from the sample in conjunction with the increase in the concentration of TSSC, resulting in a gradual decrease in the freezing point depression as the cycles progressed [39]. Regarding the freezing rate, the rates were of approximately 7.8, 7.2, 6.7, and 6.3 μm/s, for AM, CC_f1_, CC_f2_, and CC_f3_, respectively. These values indicate moderate freezing rates, and it is an expected result due to the progressive reduction in the water content of the samples, with a gradual increase in TSSC values during the cycles. Hence, as TSSC concentration increased throughout the cycles, longer times were required for the samples to reach the freezing of the liquid sample, since the solutes interfere with the formation and growth of ice crystals [49]. In this context, the solutes hinder the organization of water molecules into a solid structure, slowing the advance of the freezing front [52]. Additionally, our values of freezing rate were lower than the critical value (≈8.0 μm/s) commonly used to differentiate freezing regimes [53]. Specifically, rates above this threshold may lead to excessively rapid freezing, and it reduces the possibilities of reaching a considerable separation of fractions due to the occlusion of solutes within the ice structure during the freezing stage [49].

Importantly, the freezing rate is known to influence the size and morphology of ice crystals. At higher freezing rates, rapid nucleation is favored, while the crystal growth is limited, producing small and uniform crystals, which make the phase separation and the subsequent extraction of the concentrated fraction more difficult. In contrast, at lower freezing rates, this allows the formation of greater crystal growth, resulting in larger ice structures that facilitate the extraction of concentrates compared with high freezing rates [39]. Therefore, the conditions used for the concentration of the AM sample could be suitable for promoting the phase separation, and in turn, it could be ideal to recover different biocomponents, enhancing the unique characteristics of the raw material.

### 3.2. Chemical Composition

Table 1 shows the chemical composition in terms of moisture, protein, and lipid contents in CAM, AM and the concentrated samples at the third cycle (CC_f3_ and Ev_3_).

For fresh AM, it presented initial moisture, protein, and lipid content values close to 92.1, 1.6, and 1.3%, respectively. These values were significantly lower than those reported by Eshonturaev et al. [54] (protein content ≈4.2%, and lipid content ≈2.6%). This behavior may be associated with multiple factors that affect the chemical composition of the almonds, including harvest conditions (time and temperature), type of extraction (manual or mechanical), edaphoclimatic conditions during almond growth (e.g., water stress, soil composition, altitude, and use of fertilizers), elaboration of almond milk (pre- and post-storage conditions), processing parameters (water-to-almond ratio, temperature, soaking, and filtration), and type of packaging [55]. The high moisture content and low protein and lipid contents observed in the CAM sample are likely related to the common formulation of commercial almond drinks, which generally contain a higher proportion of water and lower protein and lipid contents due to their low almond percentages, as well as additional ingredients such as maltodextrin, vegetable oils, stabilizers, and emulsifiers. In the present study, our CAM sample contained only 3% of almond paste. Hence, the relatively low almond content could explain the reduced protein and lipid levels compared with the experimental AM sample. Additionally, the CAM sample exhibited the highest moisture content, corroborating its more diluted composition typical of conventional commercial almond beverages.

Once the concentration treatments were applied, the moisture content value decreased in all concentrated samples, indicating that both CBFC and evaporation processes were effective in water removal, although evaporation resulted in a more pronounced reduction. In contrast, the protein and lipid contents presented the highest values after the CBFC treatment. These behaviors suggest that CBFC treatment reduces the risk of denaturation and protein aggregation [56], as well as limits phenomena such as phase separation, thermal oxidation and interactions with denatured proteins in lipids [57]. In the same way, Hwang et al. [58] reported a significant increase in the concentration of proteins and lipids in milk treated by FC and evaporation processes. However, the results did not show significant differences between the treatments. Thus, the differences observed in the present study may be associated with the specific operating conditions used in each process (e.g., temperature, processing time, and degree of water removal), which can influence the stability and retention of various macronutrients. Therefore, under the conditions evaluated in this study, the CBFC treatment showed a higher concentration of protein and lipids compared with the evaporation process. This tendency may be attributed to the low temperatures used during the CBFC process, which are generally considered advantageous for heat-sensitive matrices such as plant-based beverages, where the stability of proteins and lipids can be affected during thermal treatment. Although Hwang et al. [58] reported similar behavior between both FC and evaporation methods, the findings of the present study suggest that the CBFC process, under appropriate conditions, may represent a favorable technology for obtaining concentrated products with improved preservation of TBC content.

### 3.3. Physicochemical Properties

The impact of CBFC and evaporation treatments on the physicochemical properties of the samples is summarized in Table 2. In both CBFC and thermal treatments, the TSSC values did not present significant statistical differences in the same cycle.

For fresh AM, it showed initial TSSC, pH, TTA, L*, a*, and b* values close to 1.4 °Brix, 6.0, 0.2 g CA/100 g, 71.1, 2.1, and 19.7 units, respectively. Specifically, the AM exhibited high lightness, indicating a relatively light color. Meanwhile, the positive a* and b* values suggested a slight tendency toward reddish and yellowish tones, respectively. Overall, the product exhibited a light appearance with a predominance of soft yellow tones.

For TSSC, pH, and TTA values, our values were lower than those reported by Manzoor et al. [23], who found values of 4.5 °Brix, 6.1, and 0.2 g CA/100 g, respectively. Similarly, for CIELab parameters, our results also differed from those reported by Yilmaz-Ersan and Topcuoglu [59], who reported CIELab values close to 62.8, −0.85, and 5.4 units, for L*, a*, and b* parameters, respectively. Specifically, variations in the physicochemical properties of fresh AM may be associated with the formulation and processing conditions during AM preparation, including the proportion of ingredients used for the preparation of soaked almond seeds (ratio of raw almond seeds and distilled water, and processing temperature) and the use of external parameters such as grinding time and the type and pore size of the filter used. Furthermore, differences in CIELab values could be connected to the light scattering effects related to the particle size (smaller particle sizes tend to present higher L* values) and the presence of suspended solids and lipids inherent to the almond (higher content may increase the intensity of the yellow color (b* axis)) [60]. Moreover, the properties of the raw almonds may vary depending on climatic and geographical factors such as temperature, type of soil, and altitude, among others [37,61,62]. These factors may partly explain the differences observed with the study of Yilmaz-Ersan and Topcuoglu [59], since their study was conducted in Turkey, whereas the present study was carried out in Chile, two countries with distinct climatic conditions.

For the CAM sample, it exhibited markedly different physicochemical properties compared with the experimental AM sample. The CAM sample presented lower TSSC, protein, lipid, and titratable acidity values, and higher moisture and lightness (L*) values than the AM sample, indicating that these differences can be directly associated with the formulation composition of CAM, where, as previously mentioned, the high amount of water used in the formulation of CAM generates a considerably more diluted liquid matrix. Specifically, the proportion of ingredients in the CAM beverages is commonly designed to improve economic feasibility, using a limited proportion of almonds in the final sample, and it can be correlated with the TSSC values, since a low concentration of almond paste was used in the CAM sample. Moreover, the higher pH and lower TTA values compared with the AM sample may be related to the buffering effect generated by stabilizers and emulsifiers, since these ingredients contribute to higher lightness values and a more homogeneous appearance by improving colloidal stability and light scattering properties within the beverage matrix [63].

Once the CBFC and evaporation treatments were applied to fresh AM, the solutes showed a progressive increase at each cycle. In the CBFC process, each cycle produced a higher concentration than the previous one, reaching values of approximately 2.7, 3.3, and 4.1 °Brix, for CC_f1_, CC_f2_, and CC_f3_, respectively. Meanwhile, for evaporation treatment, the concentration values were close to 2.8, 3.3, and 4.0 °Brix, for Ev_1_, Ev_2_, and Ev_3_, respectively. Thus, although slight differences in mean °Brix values were observed in some cycles, there were no significant differences between the corresponding cycles. Therefore, both CBFC and evaporation processes resulted in comparable final concentration levels, allowing a fair comparison of quality attributes.

For CBFC treatment, this trend may be associated with the axial heat transfer and the moderate freezing process. These conditions favor a better phase separation and promote a counter-diffusion phenomenon. Moreover, as the temperature progressively decreased up to −20 °C, the concentrated solute molecules managed to separate from the ice/water molecules, which may facilitate the extraction process. To date, there are no studies on the application of BFC or CBFC treatments applied to almond milk. However, a progressive increase in solute concentration has been widely reported in many food matrices such as acerola pulp [33], milk [34], cheese whey [64], extracts (peppermint (*Mentha piperita*) [35,36,38], mate (*Ilex paraguariensis*) [65], green tea (*Camellia sinensis*) [66], and coffee [67]), and fruit juices (pomegranate [68], strawberry [69], and maqui and calafate [70], among others). Hence, under appropriate BFC conditions (axial heat transfer at −20 °C), an effective phase separation may be achieved, where the isolated solutes from the ice fraction can reach a high concentration. However, further studies evaluating different BFC conditions with a greater number of cycles are required.

In pH and TTA terms, opposite trends were observed as the cycles progressed, since pH showed a significant downward trend, whereas TTA showed a significant upward trend. The pH and TTA values ranged from 5.7 to 5.2 and from 0.4 to 1.0 g CA/100 g, from the first cycle to the final cycle, respectively. This contrary behavior has been connected with the significant increase in solutes, since as the solute concentration increased cycle to cycle, the organic acid content increased during the cycles [71]. Similar trends between pH and TTA have been reported in other liquid foods treated by BFC such as fruit juice [32], wine [49], and skimmed milk [72], showing the consequence of BFC technology on solute concentration and its subsequent effects on key physicochemical properties relevant to food processing.

For CIELab parameters after the CBFC process, the L* and a* values significantly decreased, whereas the b* value significantly increased as the cycles advanced, reaching L* = 57.3, a* = −0.5, and b* = 29.9, respectively. These results indicate a progressive loss of luminosity cycle by cycle, along with a slight change in the a* axis toward negative values, suggesting a transition from a slightly reddish tone to a slightly greenish tone. At the same time, the b* value considerably increased, evidencing a more pronounced yellow tone. This behavior can be related to the considerable increase in solutes and the corresponding reduction in the amount of water during the cycles. Consequently, the final cycle presented higher solute levels and lower water content than the sample from the first cycle [73]. Similar trends have been reported in liquid foods treated by BFC technology such as fruit juices [28,69] and extracts [35,36], where all the samples were darker in comparison with the original sample, with dissimilar behaviors in a* and b* values. These results suggest that the BFC treatment can preserve the color of the original sample, since the application of thermal treatment affected the luminosity and yellow hue after three cycles at high temperatures (L* = 41.1, a* = 0.9, and b* = 37.7), which may be associated with the degradation of certain components that influence the color of liquid foods [49].

For ΔE* terms (it was compared with the AM sample), the values increased with each cycle, reaching 17.2, 22.9, and 27.7 units, for CBFC, and 20.0, 25.7, and 33.0 units, for evaporation, for the concentrate fractions in C_1_, C_2_, and C_3_, respectively. This increase can be correlated with the rise in TSSC values, since a higher solute concentration during the cycles indicates that higher ΔE* value can be obtained [29]. Specifically, thermal treatment produced more pronounced color changes than the CBFC process. It suggests a more pronounced chromatic impact, possibly associated with the degradation of biocomponents at high temperatures [74]. According to Krapfenbauer et al. [75], ΔE* values higher than 3.5 units indicate clearly perceptible differences by the human eye between a treated sample and the original sample. In our case, all the concentrated samples could be visually differentiated from the fresh AM. Hence, both CBFC and evaporation treatments exhibited ΔE* values well above this threshold, confirming that the changes in color induced by the concentration processes are easily detectable with the naked eye. Similar behavior has been observed in other liquid foods concentrated by BFC, where this technology produces less severe alterations in the color due to the lower degradation of biocomponents than that observed in samples treated by high-temperature technologies [25,26].

### 3.4. TBC Content and Antioxidant Activity

**Figure 3:**
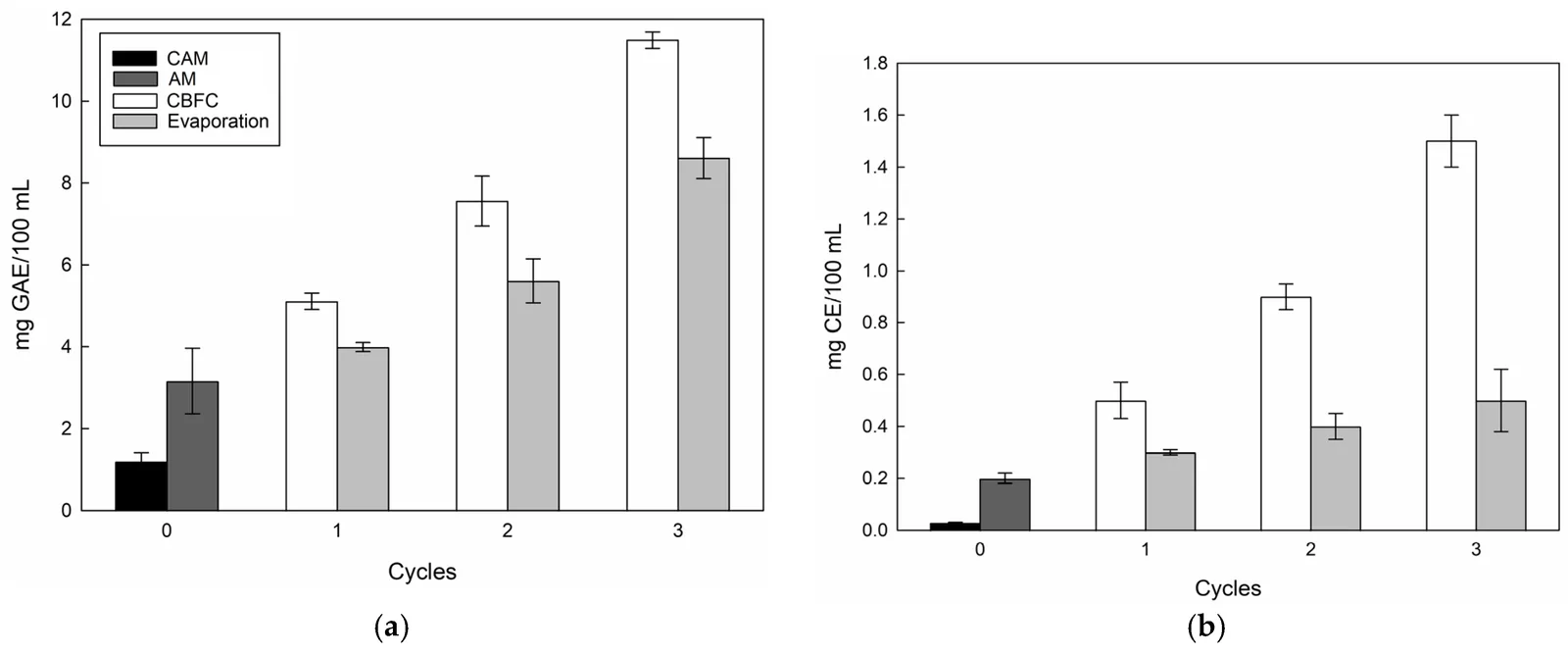

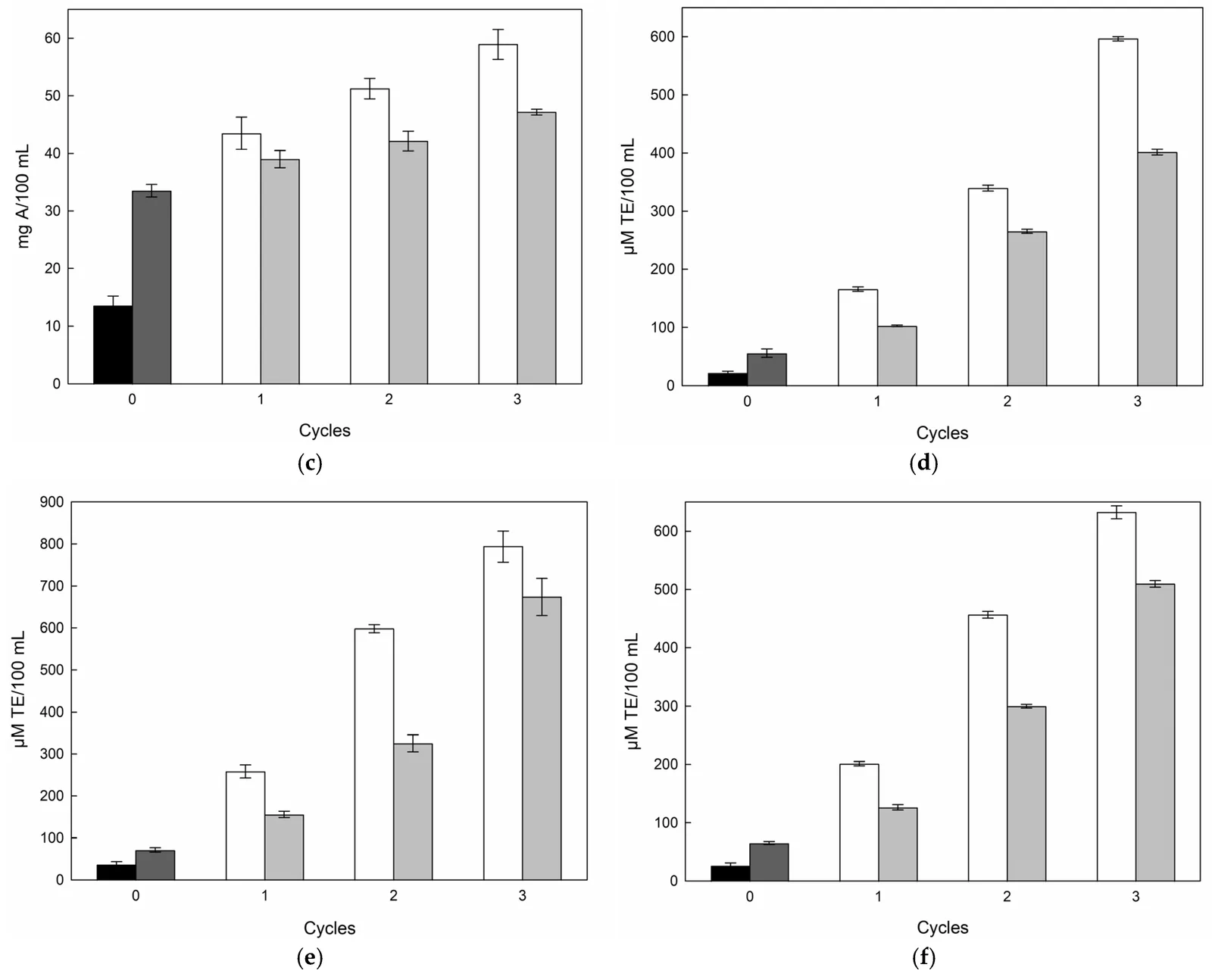
TBC content and antioxidant activity of CAM, AM and concentrated samples: (**a**) Total phenolic content (TPC); (**b**) total flavonoids content (TFC); (**c**) protein content (PRC); (**d**) 2,2-diphenyl-1-picrylhydrazyl assay (DPPH); (**e**) ferric reducing antioxidant power assay (FRAP); and (**f**) oxygen radical absorbance capacity assay (ORAC).

The effect of CBFC and evaporation treatments on the TPC, TFC, PRC, DPPH, FRAP, and ORAC in CAM, AM and the concentrated samples is summarized in Figure 3.

The fresh AM exhibited TPC, TFC, PRC, DPPH, FRAP, and ORAC values close to 3.16 mg GAE/100 mL, 0.2 mg CE/100 mL, 33.5 mg A/100 mL, 55.9, 71.38, and 65.11 µM TE/100 mL, respectively. In comparison with other studies, our TPC, TFC, PRC and DPPH values were significantly different than those found by La Torre et al. [76] and Gülhan [77], who observed values of approximately 16.5 mg GAE/100 mL, 0.7 mg CE/100 mL, 0.4 mg/100 mL, and 23.3 μM TE/100 mL, and 220 mg GAE/g, respectively. The increase or decrease in the TBC content and antioxidant activity can be connected with the variations in climatic conditions during the growth stage of the almonds. In this study, our almonds were harvested in south-central Chile, a region with a temperate Mediterranean climate influenced by oceanic conditions, which is characterized by dry and warm summers, with cool and rainy winters [78]. Meanwhile, Bologna has a temperate continental climate, with warm and humid summers, and cold winters, often with frost and occasional snow, as well as a marked annual temperature range with distributed precipitation, although with a maximum in winter and autumn [79]. Additionally, there are other factors that can influence the characteristics of the almonds such as cultivation practices, maturity of the plant during harvest, post-harvest storage, pre- and post-harvest infections, and edaphoclimatic conditions (water stress, soil composition, altitude, and temperature, among others, which can affect the formation of secondary metabolites) [55]. These factors can lead to almonds with distinct properties, reflecting in their color, flavor, and various internal properties, including chemical composition, physicochemical properties, TBC content and antioxidant activity. Furthermore, the different processing conditions used in the preparation of fresh AM can cause a significant variation in its properties [12,13]. Additionally, the higher TBC content and antioxidant activity values observed in the fresh AM sample may be associated with higher almond solids content and lower degree of formulation dilution in comparison to the commercial almond beverage. Specifically, in our case, we used fresh almonds, whereas the CAM sample was made with almond paste (3%, without details of preparation). Therefore, as many phenolic compounds and antioxidant activity of almond beverages primarily originate from almond fruits, the formulations with increased almond concentrations are expected to exhibit a greater reducing power and radical scavenging activity. Thus, the CAM products are commonly formulated with low almond percentages and multiple stabilizing additives, whereas our experimental AM sample was prepared with fresh almonds under controlled laboratory conditions, favoring greater retention of naturally occurring almond constituents. Moreover, from a technological perspective, the results demonstrate that the experimental AM sample provided a more bioactive-rich matrix than the commercial formulation, establishing a favorable baseline for subsequent concentration treatments.

Therefore, in both CBFC and evaporation techniques, the TBC content and antioxidant activity values significantly increased as the cycles advanced. The highest values were achieved in CC_f3_, with 1.8-, 7.5-, 3.6-, 10.7-, 11.1-, and 9.7-fold increases versus 1.4-, 2.5-, 2.7-, 7.2-, 9.4-, and 7.8-fold increases, with respect to the fresh AM values, for TPC, TFC, PRC, DPPH, FRAP, and ORAC, respectively. In particular, the low-temperature technology resulted in a significant increase in the concentration of the analyzed biocomponents. This effect may be attributed to the extraction of the concentrated solution in synergy with the partial removal of water during the cycles [80]. Consequently, CBFC technology produces higher values than the high-temperature technology. Moreover, this behavior may be associated with the TSSC values, where, as the cycles increased, the TSSC values increased, and thus, the TBC content and antioxidant activity values increased, indicating that TBC content and antioxidant activities tended to increase as TSSC increased. Furthermore, an important factor is the acidity, since as the acidity increases, the bonds within the cell walls are broken, and thus, it is possible to facilitate the conversion of phenolic compounds into free forms (aglycones), leading to an increase in the concentration of polyphenols [81]. Similar upward trends in TBC content and antioxidant activity have been reported in various liquid foods treated by FC such as wine [49], milk [82], coffee [83,84], *Centella asiatica* [85] and broccoli [86] extracts, apple juice [87], and tofu [88]. Hence, these studies suggest an association between concentration technologies, solutes, and biocomponents content, since the application of FC or evaporation produces a continuous increase in TBC content and antioxidant activity. However, in all cases, FC technology showed higher values than evaporation. This behavior may be associated with the low-temperature processing conditions, which are generally reported as a factor that reduces the degradation of biocomponents compared with high-temperature technologies [68,70].

As previously mentioned, the TBC content and antioxidant activity values obtained after the thermal treatment were lower than those observed after the CBFC treatment. This difference could be related to the temperature of concentration used during the evaporation treatment (50 °C), since the elevated temperature accelerated the oxidation of phenolic compounds, decreasing the retention of TBC content and antioxidant activity in the final sample [89]. In addition, for the PRC assay, the high temperatures may also favor the protein denaturation [90], which could explain the higher degree of denaturation observed in each sample treated at 50 °C compared to those processed by CBFC treatment, reinforcing the fact that the low temperatures limit the oxidation/denaturation of relevant bioactive compounds. Overall, the results suggest that the application of low temperatures during the concentration process may contribute to low degradation of thermolabile compounds as the cycles advanced [91]. Nevertheless, future studies should be focused on the degradation kinetics and compound-specific stability to fully confirm the effects of CBFC process at each cycle.

Therefore, our results demonstrate under the evaluated conditions that CBFC technology is an alternative non-thermal technology to concentrate various liquid foods, including plant-based milk foods.

### 3.5. Sensory Evaluation

**Figure 4:**
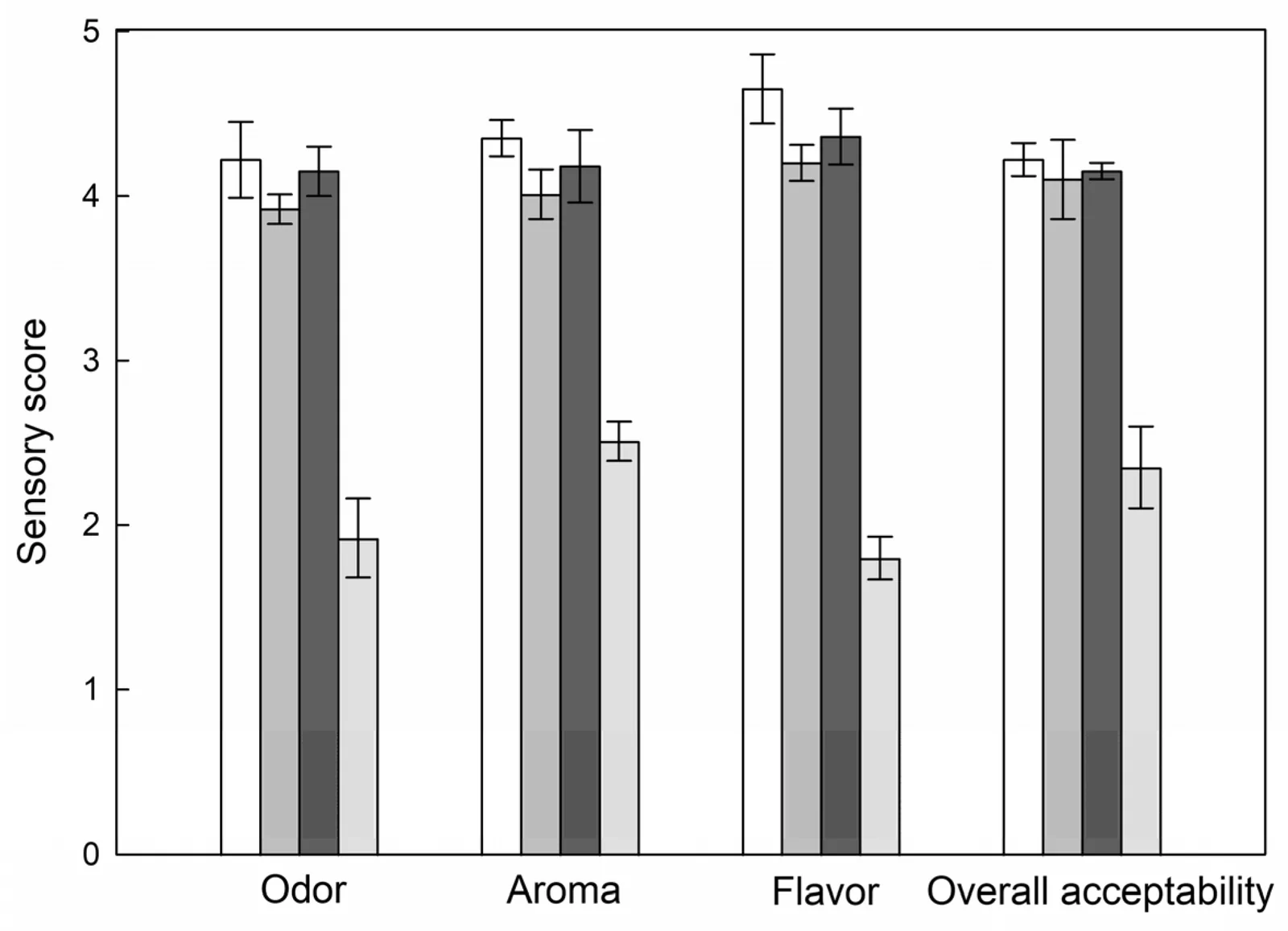
Sensory evaluation (odor, aroma, flavor and overall acceptability) of CAM (white rectangle), AM (gray rectangle), and reconstituted concentrated samples by CBFC (black rectangle) and evaporation (light gray rectangle) (third cycle) treatments.

The acceptance scores attributed by the panelists between CAM, AM, and reconstituted concentrated (third cycle) samples are indicated in Figure 4.

Firstly, for all the sensory attributes, no statistically significant differences (*p* > 0.05) were found between CAM, AM and the reconstituted concentrate obtained by CBFC technology. The sensory scores showed that the CAM sample presented the highest sensory scores for all evaluated attributes in comparison with the other samples. Descriptively, the sensory attributes of the reconstituted cryoconcentrate sample remained within the same range observed for the non-concentrated samples, all with sensory scores above 4 points. These values indicate that the samples maintained a generally favorable sensory perception and relatively similar acceptance among the panelists. In contrast, the lowest scores were observed for the evaporation sample, showing significant differences (*p* ≤ 0.05) compared with the other samples. Hence, this sample exhibited a marked reduction in sensory perception, especially for odor and flavor, where scores decreased below 2.0 points.

The values (without statistically significant difference) between the CAM, AM, and the reconstituted cryoconcentrate samples indicate that the CBFC technology preserves the original sensory characteristics of the AM sample, whether from the commercial product or the freshly prepared formulation. This behavior may be associated with the non-thermal nature of FC treatment, where the water removal occurs through ice-crystal formation at low temperatures, minimizing the degradation of heat-sensitive compounds such as volatile molecules responsible for sensory perception [92].

These results were similar to studies on FC applied to blackcurrant juices [93] and Andes berry pulp [94], where the respective cryoconcentrated products showed sensory characteristics comparable to those of the fresh sample for the same attributes analyzed in the present study. Moreover, Moreno et al. [67] demonstrated a high retention of sensory (volatile compounds) qualities of coffee extract samples subjected to BFC and falling-film FC treatments. Hence, FC is an advantageous technology from a sensory and organoleptic viewpoint, since it has not only demonstrated the retention of sensory characteristics in juices and fruit pulp, but also in plant-based milk alternatives like almond milk. Additionally, the significant sensory differences observed with the evaporated sample may be attributed to the thermal stress generated during the evaporation treatment. Specifically, heat treatments can promote lipid oxidation, Maillard reactions, protein denaturation, and the formation or loss of volatile compounds, leading to undesirable cooked or oxidized flavors and negatively affecting panelist perception [95]. Therefore, these results suggest the advantage of the CBFC technique in concentrating liquid foods, in particular for the retention of the sensory characteristics of the original sample. Accordingly, the reconstituted cryoconcentrate shows potential for future commercialization studies.

### 3.6. Process Parameters

The effect of CBFC on the process parameters, such as CI, Eff_s_, and Sy in each cycle, is summarized in Table 3.

For CI, the values progressively increased with each cycle, reaching CI values of 1.9, 2.4, and 2.9, for C_1_, C_2_, and C_3_, respectively. The CI values suggest that a lower amount of solutes was lodged in the IC_f_ (a phenomenon commonly referred to as the occlusion phenomenon) as the cycles advanced [47,96]. However, our results were lower than those reported in previous FC studies, which observed CI values of 33.9, 17.1, and 7.0, in the last cycle, for peppermint infusion treated by centrifugal-percolation BFC [38], whey protein under elution-centrifugal BFC [97], and noni tea processed by gravitational-BFC [98], respectively. These differences may be associated with the separation strategy, since the present study used centrifugation as the only external force, whereas previous studies applied additional external forces during the separation process or used liquid matrices with easier mobility through ice channels. Even so, the centrifugation allows the extraction of highly concentrated solutes from the IC_f_. However, it is necessary to study the application of two external forces at the same time for the fraction separation, together with different freezing temperatures and various centrifugation conditions.

For Eff_s_, the results were of approximately 82.5, 78.6, and 74.7%, for C_1_, C_2_, and C_3_, respectively. The high Eff_s_ values can be attributed to the low TSSC value of the initial sample (fresh AM) as well as the concentrated fraction at each cycle, resulting in a low viscosity. Thus, these conditions may facilitate the mobility of the solutes from the IC_f_ to the outside once centrifugal force was applied. However, as the cycles progressed, a notable decrease in Eff_s_ values was observed, since the concentrated solution used as the stock solution had a higher viscosity than the previous stock solution. Therefore, although the viscosity was suitable for solute transport through the ice channels, the increase in viscosity in each cycle hindered the movement of solutes in the extraction process [99]. Our Eff_s_ results are comparable to those reported for other liquid foods treated by BFC, e.g., Adorno et al. [69] reached 74% during the concentration of strawberry juice. However, our values were lower than those reported in studies that incorporated an external force into the CBFC process, since Pérez-Bermudez et al. [38], and Bastías-Montes et al. [100] obtained Eff_s_ values of approximately 95 and 96%, using percolation-CBFC and filtration-CBFC, respectively. Hence, the FC technology is often reported to be influenced by factors such as solute concentration and viscosity, since high TSSC values lead to a high viscosity, and this combination reduces the Eff_s_ value. Additionally, this process parameter can be significantly increased if an external force is added to the conventional CBFC system [101].

For S_y_, the values increased as the cycles advanced, with S_y_ values of 38.9, 51.4, and 72.1, for C_1_, C_2_, and C_3_, respectively. This trend suggests that the separation conditions enabled the recovery of a considerable amount of solutes from the ice fraction in each cycle. Hence, the S_y_ parameter may be associated with the m_f_, and even with the TSSC [29]. Thus, as the cycles progressed, the accumulation of m_f_ between the ice crystals increased, which may also be related to the viscosity at each cycle. Consequently, the increase in TSSC and m_f_ was accompanied by a significant rise in S_y_ values. Our S_y_ values were higher than those reported in previous studies under similar conditions. For example, Meneses et al. [66] and Dantas et al. [102] reported S_y_ values ranging from 18 to 67% and 68 to 79%, in green tea extract and lactose-free milk under BFC treatment, respectively. Meanwhile, Pérez-Bermúdez et al. [38], using a double-separation system in the BFC process, reported S_y_ values between 86 and 91%. These differences highlight the potential influence of multi-stage separation in liquid food samples treated by FC technology, which may contribute to increasing various process parameters, resulting in a final concentrated sample with low water content and a pure IC_f_. Therefore, a possible association between S_y_ and Eff_s_ may be observed, since the increase in mass generates a more complex flow movement of solutes toward the outside, which may contribute to the decrease in Eff_s_ values cycle by cycle [103,104], suggesting important interactions among key process parameters [105].

## 4. Conclusions

In the present study, we have established the potential of CBFC technology over three cycles as a non-thermal concentration tool applied to plant-based milk (almond milk) in comparison with the traditional thermal technology (evaporation), and this potential can be explained by the low concentration temperatures. Therefore, the level of concentration of biocomponents in the CBFC treatment was higher in comparison with the evaporation treatment due to the retention of thermosensitive bioactive compounds as the cycles progressed. Moreover, CBFC achieved a moderate separation of fractions, since CI, Eff_s_, and S_y_ showed values close to 2.9 units, 75, and 72%, respectively, and it can be explained by the increase in viscosity as the cycles advanced, and even the present study used a narrow range of separation parameters. Thus, the results obtained suggest that CBFC is a promising technology to concentrate a liquid food like almond milk, indicating a high concentration of solutes and compounds of interest, with high levels of IC_f_ purification, and in turn, this technology maintains the physicochemical and TBC content characteristics of the original product. Moreover, the sensory evaluation suggests that the CBFC technique allows the retention of many sensory characteristics from the original sample. Therefore, the reconstituted cryoconcentrate sample is an excellent option for future commercialization studies. CBFC could be applied to the development of plant-based beverages with greater added value such as products with higher TBC content density, better stability, or differentiated sensory profiles than current products on the market, without resorting to intensive heat treatments.

From an industrial perspective, CBFC technology may represent a promising alternative in line with current trends toward minimal processing and preservation of functional ingredients in plant-based matrices. This technology could contribute to product diversification and process optimization, while helping to maintain the quality attributes of the original matrix. However, further studies addressing process scalability, economic feasibility, and sensory acceptance will be necessary to fully assess its viability at an industrial level. Additionally, the concentrated fractions obtained by CBFC technology may offer potential applications in food systems and, more broadly, in nutraceutical or cosmetic formulations.

Thus, the following stages should be focused on other conditions of extraction through external force (over three centrifugation cycles), the use of different freezing temperatures (between 0 to −20 °C), and even the integration of a second external force before or after the centrifugation stage (filtration, vacuum, or percolation) to enhance the separation step. Additionally, future studies should evaluate individual compounds through more specialized techniques (HPLC analysis), revealing a clearer profile of the effects of the non-thermal concentration technology known as freeze concentration.

## Figures and Tables

**Table 1:** The effect of CBFC and evaporation on the chemical composition of CAM, AM and concentrated samples at third cycle.

Sample	Moisture Content (%)	Protein Content (%)	Lipid Content (%)
CAM	98.2 ± 1.1 ^a^	0.7 ± 0.2 ^a^	1.0 ± 0.2 ^a^
AM	92.1 ± 3.5 ^b^	1.6 ± 0.1 ^b^	1.3 ± 0.1 ^ab^
CC_f3_	80.1 ± 2.2 ^c^	5.2 ± 0.4 ^d^	3.3 ± 0.3 ^d^
Ev_3_	62.0 ± 3.7 ^d^	4.4 ± 0.1 ^c^	2.5 ± 0.1 ^c^

All data are mean ± SD. Different letters in the same column indicate significant differences. CAM: commercial almond milk; AM: almond milk; CC_f3_: concentrated samples obtained by CBFC at the third cycle; Ev_3_: concentrated samples obtained by evaporation at the third cycle.

**Table 2:** The effect of CBFC and evaporation on the physicochemical properties of CAM, AM, and concentrated samples.

Sample	TSSC (°Brix)	pH	TTA (g CA/100 g)	L*	a*	b*	ΔE*
CAM	0.8 ± 0.1 ^a^	6.4 ± 0.0 ^a^	0.1 ± 0.0 ^a^	82.4 ± 1.3 ^a^	0.1 ± 0.0 ^a^	11.2 ± 0.7 ^a^	-
AM	1.4 ± 0.1 ^b^	6.0 ± 0.0 ^b^	0.2 ± 0.0 ^b^	71.1 ± 1.8 ^b^	2.1 ± 0.1 ^b^	19.7 ± 1.1 ^b^	-
CC_f1_	2.7 ± 0.0 ^c^	5.7 ± 0.1 ^c^	0.4 ± 0.1 ^c^	66.8 ± 1.0 ^c^	0.9 ± 0.0 ^c^	22.4 ± 0.8 ^c^	17.2 ± 0.7 ^a^
CC_f2_	3.3 ± 0.0 ^d^	5.5 ± 0.0 ^d^	0.5 ± 0.0 ^cd^	63.5 ± 0.8 ^d^	0.6 ± 0.1 ^d^	25.6 ± 1.3 ^d^	22.9 ± 0.4 ^b^
CC_f3_	4.1 ± 0.0 ^e^	5.2 ± 0.3 ^de^	1.0 ± 0.2 ^e^	57.3 ± 0.1 ^e^	−0.5 ± 0.0 ^e^	29.9 ± 1.0 ^e^	27.7 ± 0.3 ^c^
Ev_1_	2.8 ± 0.1 ^c^	5.9 ± 0.2 ^bc^	0.5 ± 0.0 ^c^	62.3 ± 0.4 ^c^	1.6 ± 0.2 ^c^	26.3 ± 1.2 ^c^	20.0 ± 0.4 ^a^
Ev_2_	3.3 ± 0.1 ^d^	5.8 ± 0.3 ^bcd^	0.7 ± 0.1 ^d^	53.7 ± 1.5 ^d^	1.2 ± 0.1 ^d^	32.6 ± 1.8 ^d^	25.7 ± 0.7 ^b^
Ev_3_	4.0 ± 0.1 ^e^	5.6 ± 0.2 ^cde^	0.8 ± 0.0 ^de^	41.1 ± 0.3 ^e^	0.9 ± 0.0 ^e^	37.7 ± 2.1 ^e^	33.0 ± 0.1 ^c^

All data are mean ± SD. Different letters in the same column indicate significant differences. CAM: commercial almond milk; AM: almond milk; CC_f3_: concentrated samples obtained by CBFC at the third cycle; Ev_3_: concentrated samples obtained by evaporation at the third cycle; TSSC: total soluble solids content; TTA: titratable acidity; L*: lightness; a*: greenness–redness axis; b*: blueness–yellowness axis; ∆E*: total color difference.

**Table 3:** The effect of CBFC on the process parameters.

Sample	CI (-)	Eff_s_ (%)	S_y_ (%)
C_1_	1.9 ± 0.1 ^a^	87.5 ± 3.2 ^a^	38.9 ± 7.0 ^a^
C_2_	2.4 ± 0.0 ^b^	78.6 ± 2.7 ^b^	51.4 ± 4.9 ^b^
C_3_	2.9 ± 0.2 ^c^	74.7 ± 4.0 ^bc^	72.1 ± 7.4 ^c^

All data are mean ± SD. Different letters in the same column indicate significant differences. C_1_: first cycle; C_2_: second cycle; C_3_: third cycle; CI: concentration index; Eff_s_: separation efficiency; S_y_: solute yield.

## Data Availability

The data presented in this study are available in the article. Further inquiries can be directed to the corresponding authors.

## Abbreviations

The following abbreviations are used in this manuscript:

FC	Freeze concentration
BFC	Block freeze concentration
CBFC	Centrifugal block freeze concentration
AM	Almond milk
CAM	Commercially almond milk
C_1_	First cycle
C_2_	Second cycle
C_3_	Third cycle
CC_f_	Concentrated fraction
IC_f_	Ice fraction
TPC	Total phenolic content
TFC	Total flavonoids content
PRC	Protein content
DPPH	2,2-diphenyl-1-picrylhydrazyl
FRAP	Ferric reducing antioxidant power
ORAC	Oxygen radical absorbance capacity
CI	Concentration index
SCC_0_	Initial solutes of the AM
SCC_f_	Solutes in the CC_f_
Eff_s_	Separation efficiency
SIC_f_	Solutes in the IC_f_
S_y_	Solute yield
m_0_	Mass of solutes of the AM
m_f_	Mass of solutes of the CC_f_
